# Anti-Cancer and Pro-Immune Effects of Lauric Acid on Colorectal Cancer Cells

**DOI:** 10.3390/ijms26051953

**Published:** 2025-02-24

**Authors:** Shiori Mori, Rina Fujiwara-Tani, Ruiko Ogata, Hitoshi Ohmori, Kiyomu Fujii, Yi Luo, Takamitsu Sasaki, Yukiko Nishiguchi, Ujjal Kumar Bhawal, Shingo Kishi, Hiroki Kuniyasu

**Affiliations:** 1 Department of Molecular Pathology, School of Medicine, Nara Medical University, Kashihara 634-8521, Japan; m.0310.s.h5@gmail.com (S.M.); rina_fuji@naramed-u.ac.jp (R.F.-T.); pkuma.og824@gmail.com (R.O.); brahmus73@hotmail.com (H.O.); toto1999-dreamtheater2006-sms@nifty.com (K.F.); lynantong@hotmail.com (Y.L.); takamitu@fc4.so-net.ne.jp (T.S.); yukko10219102@yahoo.co.jp (Y.N.); nmu6429@yahoo.co.jp (S.K.); 2 Department of Cancer Biology, Institute of Biomedical Science, Kansai Medical University, Osaka 573-1010, Japan; 3 Research Institute of Oral Science, School of Dentistry at Matsudo, Nihon University, Matsudo 271-8587, Japan; bhawal2002@yahoo.co.in; 4 Department of Pathological Diagnosis, Nozaki Tokushukai Hospital, Daito 574-0074, Japan

**Keywords:** lauric acid, colorectal cancer, oxidative stress, energy metabolism, anti-cancer immunity

## Abstract

Lauric acid (LAA) is a 12-carbon medium-chain fatty acid that reportedly has antitumor and muscle-protecting effects. However, the details of these antitumor effects remain unclear. Therefore, in this study, we investigated the mechanism underlying the antitumor effects of LAA in CT26 and HT29 colorectal cancer (CRC) cell lines. Our in vitro findings demonstrated that LAA suppressed CRC cell proliferation, induced mitochondrial oxidative stress (reactive oxygen species (ROS)), inhibited oxidative phosphorylation (OXPHOS), and induced apoptosis. Moreover, in vivo analysis of LAA showed a more pronounced antitumor effect in CT26 cells in a syngeneic mouse tumor model than in vitro; therefore, we further investigated its impact on host antitumor immunity. We observed that LAA increased the number of effector T cells in mouse tumors, while in vitro LAA activated mouse splenocytes (SplC) and promoted OXPHOS. In two-dimensional co-culture of SplC and CT26 cells, LAA induced cell death in cancer cells. In three-dimensional co-culture, LAA promoted SplC infiltration and suppressed the formation of tumor spheres. Thus, LAA may exert antitumor effects through increased ROS production in cancer cells and effector T cell activation via increased energy metabolism. These results suggest that LAA, when used in combination with existing anti-cancer drugs, is likely to exhibit sensitizing effects in terms of both antitumor and antitumor immune effects, and future clinical studies are anticipated.

## 1. Introduction

In 2020, colorectal cancer (CRC) was the third most common cancer globally, with over 1.9 million cases and 930,000 deaths [1,2]. With population aging, CRC cases and deaths are projected to reach 3.2 million and 1.6 million by 2040, making it a serious global health problem [3]. In response to this, in addition to radical surgical therapy, radiation therapy, chemotherapy, and immunotherapy are selected depending on the stage of CRC. In particular, combination chemotherapy of FOLFOX (5-FU + Leucovorin + oxaliplatin), FOLFIRI (5-FU + Leucovorin + CPT-11), CapeOX (capecitabine + oxaliplatin), and FOLFOXIRI (FOLFIRI + oxaliplatin + bevacizumab or anti-EGFR antibody) has been established as the standard treatment. In addition, immune checkpoint inhibitors are used for MSI-High cases. Despite advances in treatment, improvements in survival rates for CRC, especially in cases with metastasis, are still poor [4,5]. The complexity and diversity of malignant CRC phenotypes have been identified as causes for the lack of improved survival [5,6]. CRC involves multiple subtypes with different cellular pathways [7]. This complexity requires customized treatments that target specific genetic mutations and cellular environments within each subtype; however, developing such precise approaches remains challenging [8,9]. For example, mutations in pathways such as RAS/RAF/mitogen-activated protein kinase complicate the efficacy of specific targeted therapies, leading to treatment resistance and limited treatment options [10,11]. Thus, the development of novel treatments based on new approaches to existing treatments is considered an urgent issue for CRC. In this context, antitumor therapies targeting mitochondrial energy metabolism in cancer cells have been attracting attention in recent years [12,13]. In particular, natural compounds have shown beneficial effects regardless of CRC subtype [14,15]. We have also reported the antitumor effects of pterostilbene and berberine [16,17,18].

Fatty acids have attracted attention as dietary nutrients owing to their antitumor effects. Compared with long-chain fatty acids (LCFAs), which have both antitumor and tumor-promoting effects, medium-chain fatty acids (MCFAs) have antitumor effects. However, MCFAs are absorbed faster in the intestine and taken up into the tissues compared with LCFAs. MCFAs enter mitochondria without the carnitine shuttle, undergo rapid β-oxidation, and boost OXPHOS. In cancer cells with mitochondrial damage, this enhances ROS production and induces cell death [19,20,21]. Moreover, MCFAs also have immune-modulating effects [22] and have attracted attention as immune nutrients in cancer and inflammatory diseases [23]. However, their specific effects compared to those of LCFAs are yet to be elucidated. In particular, lauric acid (LAA) has garnered attention for its preventive effects against cardiovascular disorders [24]. In the context of its antitumor properties, studies have reported that LAA reduces tumorigenicity [25], inhibits tumor growth [26], and suppresses epidermal growth factor receptor signaling [27]. Additionally, it has been shown to modulate microRNA and long non-coding RNA activity [28,29,30], influence energy metabolism reprogramming [20], and suppress peritoneal dissemination in mice [31,32]. Furthermore, its role in reversing drug resistance in pancreatic cancer [19,33] has gained increasing interest in recent years. However, previous research on the impact of LAA on antitumor immunity remains limited Furthermore, the clinical application of MCFA to CRC remains a challenge for the future. In this study, we aimed to clarify the antitumor effects of LAA, an MCFA, on CRC cells in terms of both its direct effect on cancer cells and its effect on the immune system and to clarify the usefulness of LAA against cancer.

## 2. Results

### 2.1. LAA Inhibits CRC Cell Proliferation

**Figure 1:**
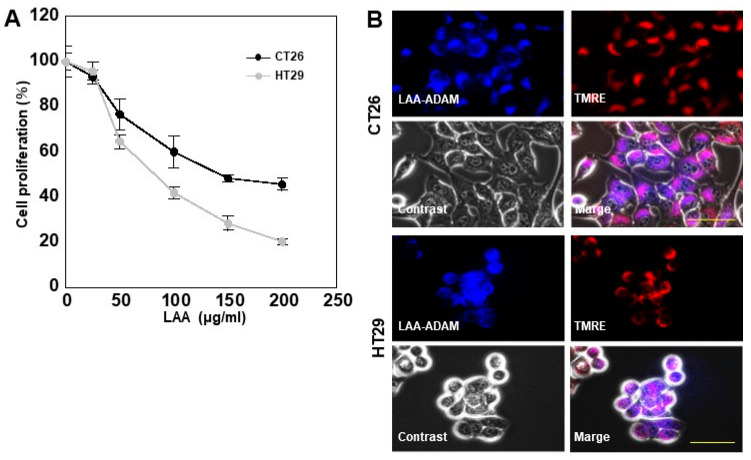
Effect of LAA on CRC cell proliferation. (**A**). LAA inhibits cell proliferation in a dose-dependent manner after 48 h. (**B**). Intracellular localization of ADAM-labeled LAA; mitochondria labeled with TMRE. Scale bar: 20 μm. Error bars: SD from three independent trials. Statistical analysis: ANOVA with Bonferroni correction. CRC, colorectal cancer; LAA, lauric acid; ADAM, 9-anthryldiazonethane; TMRE, tetramethylrhodamine ethyl ester; ANOVA, analysis of variance.

LAA administration revealed a dose-dependent inhibition of proliferation in CT26 and HT29 colon cancer cells (Figure 1A). Growth inhibition was more pronounced in HT29 than in CT26 cells. Intracellular localization of fluorescently labeled LAA showed its co-localization with tetramethylrhodamine ethyl ester (TMRE). These fluorescenes were marged, suggesting LAA localization in the mitochondria (Figure 1B). These results suggest that LAA inhibited cell proliferation by affecting mitochondria.

### 2.2. LAA Decreases Mitochondrial Function

**Figure 2:**
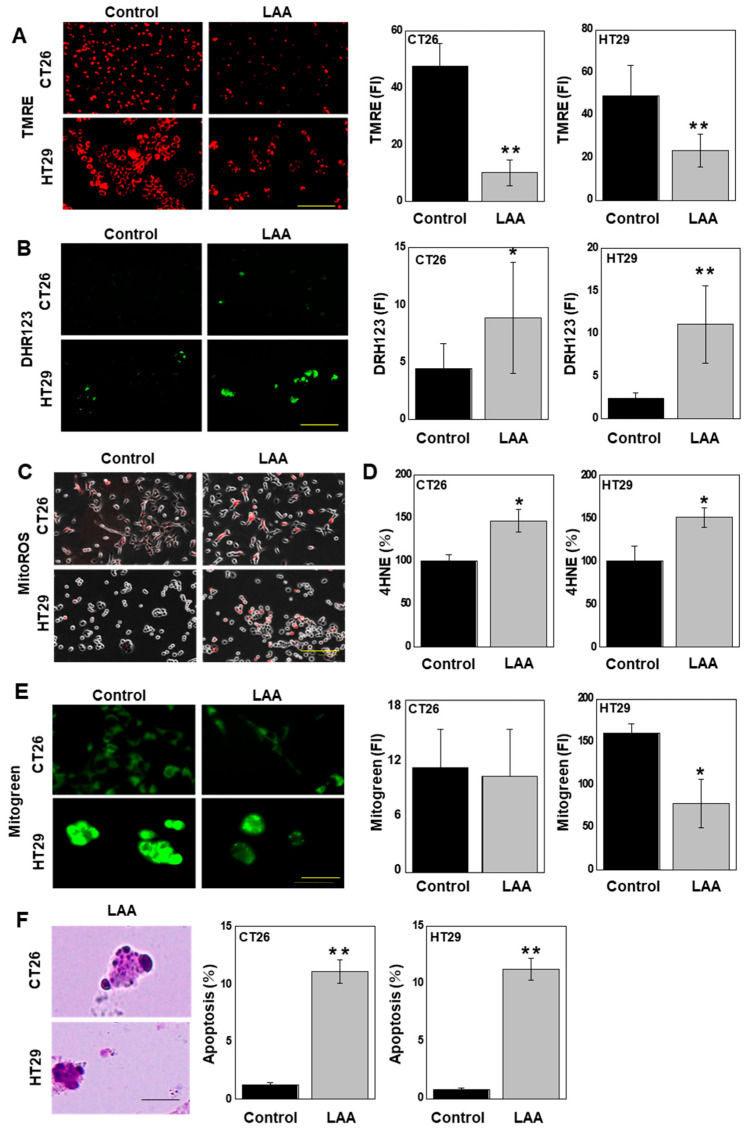
Effect of LAA on mitochondrial function. CRC cells were treated with LAA (40 μg/mL = IC_20_) for 48 h. (**A**). Assessment of mitochondrial membrane potential using TMRE. (**B**). Measurement of mitochondrial H_2_O_2_ levels using DHR123. (**C**). Assessment of mitochondrial superoxide using MitoSOX. (**D**). Assessment of lipid peroxide using 4HNE. (**E**). Measurement of mitochondrial volume using mitogen staining. (**F**). Detection of apoptotic bodies in 1100 cells stained with Giemsa dye. Right panels: semi-quantitative fluorescence analysis. Scale bar: 50 μm. Error bars: SD from three independent trials. Statistical analysis: ANOVA with Bonferroni correction. * *p* < 0.05, ** *p* < 0.001. CRC, colorectal cancer; LAA, lauric acid; TMRE, tetramethylrhodamine ethyl ester; DHR123, dihydrorhodamine 123; 4HNE, 4-hydroxynonenal; MitoSOX, mitochondrial superoxide; ANOVA, analysis of variance.

As LAA is localized in the mitochondria, we next examined the effect of LAA on mitochondrial function (Figure 2). Mitochondrial membrane potential, as measured using TMRE, decreased in both cell lines (Figure 2A). Next, to examine ROS production in the mitochondria, mitochondrial-derived hydrogen peroxide levels, as assessed using dihydrorhodamine 123 (DHR123) (Figure 2B) and superoxide (Figure 2C), were measured by fluorescent staining. Both cell lines showed significantly increased hydrogen peroxide and superoxide levels following LAA treatment. Furthermore, the level of the lipid peroxide 4-hydroxynonenal (4HNE), which is mainly generated by hydroxyl radicals, was increased following LAA treatment (Figure 2D). LAA decreased the number of mitochondria in HT29 cells but had no effect in CT26 cells (Figure 2E). Furthermore, both cell lines showed an approximately 10-fold increase in apoptosis frequency following LAA treatment (Figure 2F).

### 2.3. LAA Suppresses Energy Metabolism in CRC Cells

**Figure 3:**
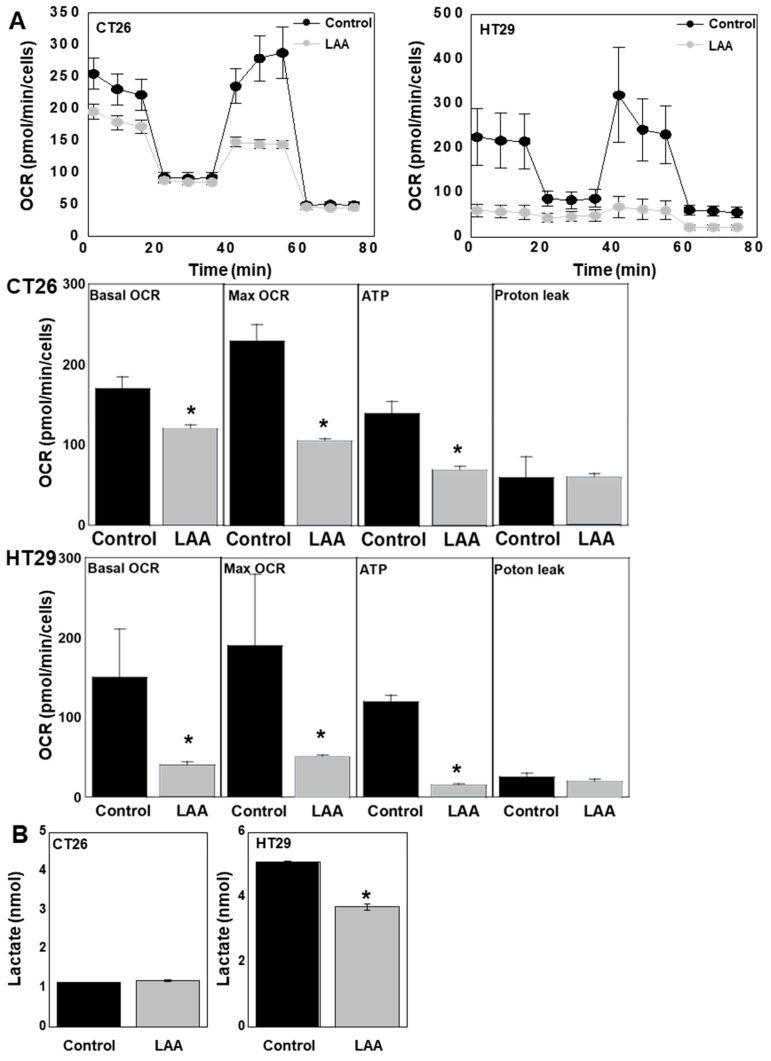
Effect of LAA on energy metabolism in CRC cells. After treatment with LAA (40 μg/mL = IC_20_) for 48 h, CRC cells were subjected to a mitochondrial stress test. (**A**). Mitochondrial respiration in the mitochondrial stress test. (**B**). Lactate concentration in the culture medium of CRC cells. Error bars: SD from three independent trials. Statistical analysis: ANOVA with Bonferroni correction. * *p* < 0.05. CRC, colorectal cancer; LAA, lauric acid; OCR, oxygen consumption rate; MAX, maximum; ANOVA, analysis of variance.

The oxygen consumption rate (OCR), measured using a flux analyzer, showed that LAA treatment reduced the respiratory quotient in both cell types (Figure 3A). LAA suppressed basal OCR, maximum OCR, and ATP production in both CRC cells. Lactate production, an indicator of glycolytic metabolism, was reduced in HT29 cells after LAA treatment; however, no significant changes were observed in CT26 cells (Figure 3B).

### 2.4. LAA Induces Changes in Stemness in CRC Cells

**Figure 4:**
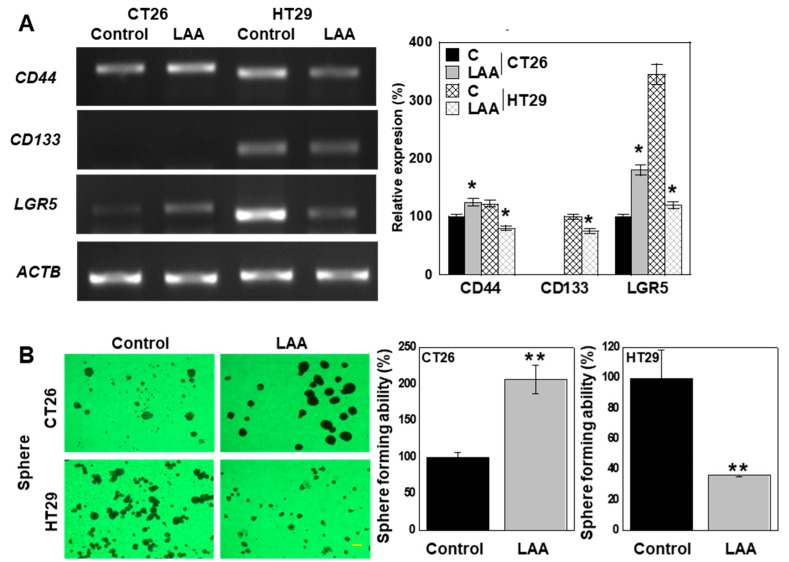
Effect of LAA on CRC cell stemness. CRC cells were treated with LAA (40 μg/mL, IC_20_) for 48 h. (**A**). Stemness-related gene expression. (**B**). Sphere formation with semi-quantification (right panel). Scale bar: 20 μm. Error bars: SD from three independent trials. Statistical analysis: ANOVA with Bonferroni correction. * *p* < 0.05 (vs. each control), ** *p* < 0.001. CRC, colorectal cancer; LAA, lauric acid; C, control; LGR5, leucine-rich repeat-containing G-protein coupled receptor 5; ACTB, β-actin; ANOVA, analysis of variance.

Next, we examined the effect of LAA on the stemness of the CRC cell lines (Figure 4) by measuring the gene expression of the stem cell markers CD44, CD133, and leucine-rich repeat-containing G-protein coupled receptor 5 (*LGR5*) after LAA treatment (Figure 4A). CT26 cells showed increased *CD44* and *LGR5* expression, while HT29 cells showed decreased expression of all stem cell markers. Stemness examined using a sphere-forming assay (Figure 4B) showed increased and decreased sphere-formation ability in CT26 and HT29 cells, respectively.

### 2.5. LAA Inhibits Peritoneal Dissemination in Mouse Colon Cancer

**Figure 5:**
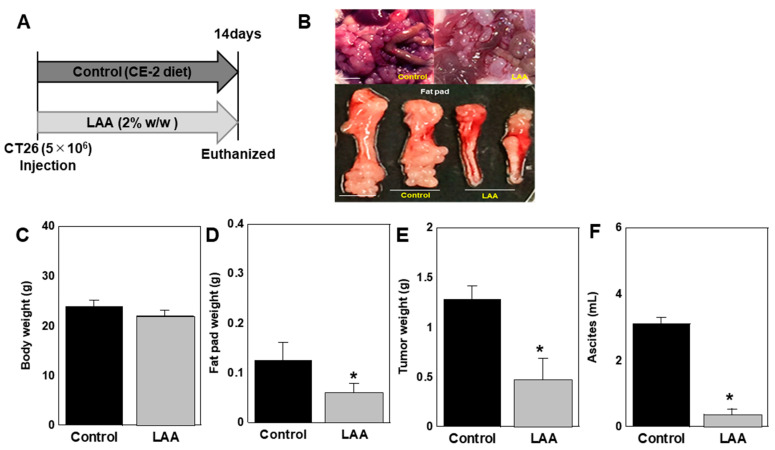
Effect of LAA on CT26 peritoneal dissemination. (**A**). CT26 cells (5 × 10^6^) were injected into the peritoneal cavity of BALB/c mice (*n* = 5). The mice were fed standard (CE-2) or LAA (2% *w*/*w*) diets. Two weeks after inoculation, the mice were euthanized. (**B**). Gross appearance of the peritoneal tumors and fat pads. Scale bar: 5 mm. (**C**). Body weights. (**D**). Fat pad weights. (**E**). Tumor weights. (**F**). Ascites volumes. Error bars: standard deviation from five mice. Statistical differences were calculated using ordinary ANOVA with Bonferroni correction. * *p* < 0.05. CRC, colorectal cancer; LAA, lauric acid; ANOVA, analysis of variance.

The effect of oral LAA intake on tumors was examined using a peritoneal dissemination model in which CT26 cells were intraperitoneally inoculated into syngeneic BALB/c mice (Figure 5). LAA was administered orally by mixing with CE-2 standard diet at 2% (*w*/*w*) (Figure 5A, Table 1). The LAA group showed no change in final body weight; however, the fat pad weight was significantly reduced (Figure 5B–D). The tumor weight and ascite volume were also significantly reduced (Figure 5E,F).

### 2.6. LAA Effects on CD8+ T Cells in SplCs

**Figure 6:**
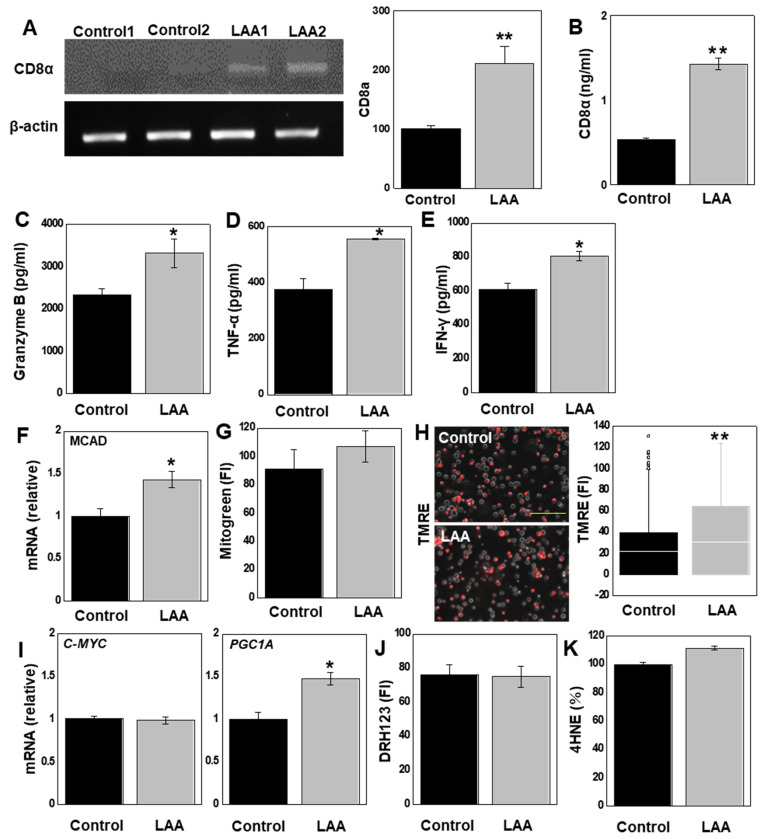
Effect of LAA on immune cells. (**A**). Analysis of immune cell properties in CT26 peritoneal tumors. Right panel: semi-quantification of RT-PCR signals. Spleen cells were collected to treat with LAA (40 μg/mL, 48 h). (**B**). mRNA expression of CD8α, a marker of effector T cells. (**C**–**E**). Cytokine concentrations in cultured medium: granzyme B (**C**), TNF-α (**D**), and IFN-γ (**E**). (**F**). *MCAD* expression. (**G**). Mitochondrial volume. (**H**). Mitochondrial membrane potential (TMRE). Right panel: semi-quantification of fluorescent intensities. (**I**). *C-MYC* and *PGC1A* expression. (**J**,**K**). Oxidative stress and mitochondrial H_2_O_2_ (**J**). Lipid peroxide (**K**). Error bars: SD from three independent trials. Statistical analysis: ANOVA with Bonferroni correction. * *p* < 0.05, ** *p* < 0.001. LAA, lauric acid; RT-PCR, reverse transcription-polymerase chain reaction; TNF, tumor necrosis factor; IFN, interferon; MCAD, medium-chain acyl-CoA dehydrogenase; TMRE, tetramethylrhodamine ethyl ester; DHR123, dihydrorhodamine 123; 4HNE, 4-hydroxynonenal; ANOVA, analysis of variance.

In the in vitro studies, LAA suppressed proliferation and increased ROS production in CT26 cells to a lower level than that in HT29 cells; however, the in vivo results showed the strong antitumor effects of LAA. We focused on the host immune system as a factor responsible for the discrepancy between the in vitro and in vivo antitumor effects (Figure 6). First, we examined the expression of *CD8α*, which indicates the infiltration of effector T cells with strong antitumor immunity. Reverse transcription–polymerase chain reaction (RT-PCR) in tumor tissue obtained from the mouse tumor model (Figure 6A) showed increased *CD8α* expression in the LAA-treated group compared with the control group (Figure 6B). Next, primary cultured mouse spleen lymphocytes (SplCs) were treated with concanavalin A (ConA) [34], which induces antigen non-specific induction of T cell blastogenesis, and then treated with LAA. The LAA-treated SplCs showed increased levels of CD8α protein, granzyme B, tumor necrosis factor (TNF)-α, and interferon (IFN)-γ released by CD8+ T lymphocytes (Figure 6B–E).

We further examined LAA-induced metabolic changes in SplCs. SplCs treated with LAA showed increased expression of medium-chain acyl-CoA dehydrogenase (MCAD) (Figure 6F). This suggests that SplCs use LAA as an energy source. Next, we examined mitochondria following LAA treatment. Although the mitochondrial volume did not change significantly (Figure 6G), we observed increased mitochondrial membrane potential (Figure 6H). Regarding energy metabolism, the expression of peroxisome proliferator-activated receptor gamma coactivator-1*α* (*PGC-1α*), an OXPHOS marker, increased, whereas we observed no significant change in *C-MYC*, a glycolysis marker (Figure 6I). SplCs treated with LAA showed no change in H_2_O_2_ or 4HNE levels and oxidative stress (Figure 6J,K).

### 2.7. Effects of LAA on Antitumor Cytotoxicity in SplCs

**Figure 7:**
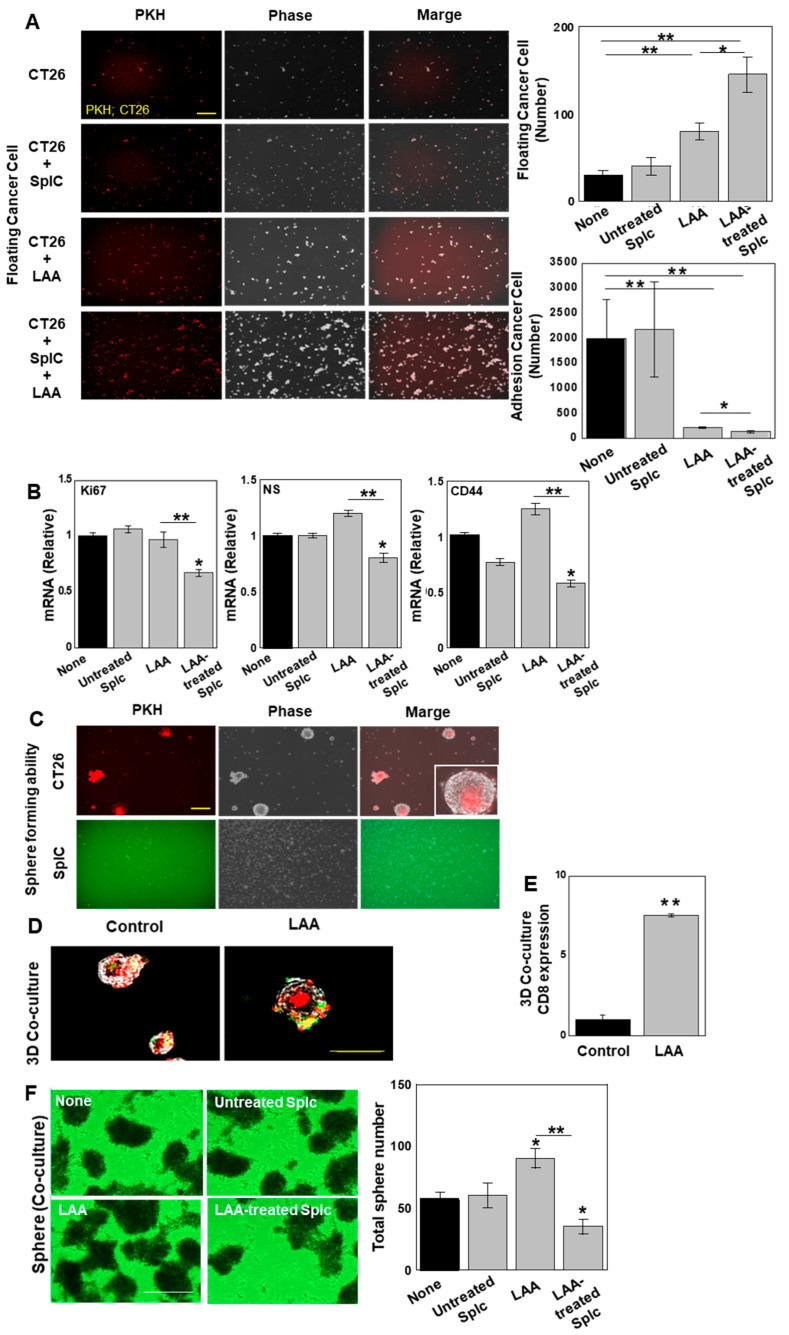
Effect of LAA on antitumoral cytotoxicity of spleen cells against CT26 CRC cells. CT26 cells (labeled with PKHred) and SplCs (labeled with PKHgreen) were subjected to 2D or 3D co-culture with or without LAA. (40 μg/mL, 48 h). (**A**). Two-dimensional co-culture of CT26 cells and SplC. Right panels: numbers of floating (upper) and adhesion (lower) cells. (**B**). Expression levels of genes related to proliferation (Ki67) and stemness (NS and CD44). (**C**). Three-dimensional co-culture of CT26 cells and SplC. (**D**). Infiltration of SplCs into CT26 spheres. (**E**). CD8α gene expression in CT26 spheres. (**F**). Sphere formation in CT26 cells co-culture with SplCs under LAA treatment. Scale bar: 50 μm. Error bars: SD from three independent trials. Statistical analysis: ANOVA with Bonferroni correction. * *p* < 0.05 (vs. each control), ** *p* < 0.001. 2D, two-dimensional; 3D, three-dimensional; CRC, colorectal cancer; LAA, lauric acid; SplCs, spleen cells; NS, nucleostemin; ANOVA, analysis of variance.

As LAA may activate CD8 + T cells in SplC, we investigated the effect of LAA on tumors induced by SplCs using an in vitro tumor model (Figure 7). First, we performed two-dimensional (2D) co-culture of SplCs with syngeneic CT26 (stained with PKHred). The number of floating cancer cells, which indicated dead cells, did not differ between the co-culture with SplCs and untfreated control. LAA alone increased floating cell number, but increased significantly when LAA treatment was added to the co-culture with SplCs (Figure 7A). The number of adherent cancer cells, which indicated living cells, did not change in co-culture with SplCs alone, but it decreased with LAA treatment alone. The number of adherent cells was further decreased in the co-culture with LAA compared to the co-culture without LAA. Additionally, the gene expression levels of *Ki67*, *CD44*, and nucleostemin (*NS*) in the CT26 cells were examined. LAA alone increased the levels of CD44 and NS; howver, co-culture of LAA-treated Splc decreased the levels of CD44, NS, and Ki67. (Figure 7B), suggesting decreased proliferation activity and stemness.

In addition, CT26 cells and SplCs were stained with PKHred and PKHgreen, respectively, to distinguish the cells and examine the effects on tumors in three-dimensional (3D) co-culture (Figure 7C). We observed sphere-forming ability in CT26 cells but not in SplCs (Figure 7C). Examination of CD8+ T cell infiltration into spheres in co-cultures of CT26 cell spheres and SplCs revealed an approximately 2.5-fold increase in the number of spheres with SplC infiltration in the LAA-treated group compared with co-culture alone (Figure 7D). Assessment of the level of infiltration revealed an approximately eight-fold increase in CD8α RNA expression in the spheres (Figure 7E). Finally, we examined the effect of the increase in SplC invasion ability by LAA on the sphere-forming ability in CT26 cells (Figure 7F). LAA alone increased CT26 sphere. However, we observed a significant decrease in the number of spheres in LAA-treated SplC co-cultures. Thus, LAA suppressed CRC cell proliferation in the 2D co-culture system, promoted lymphocyte infiltration into spheres, and suppressed sphere formation in the 3D co-culture system.

## 3. Discussion

MCFAs have demonstrated anti-cancer effects, promoting cancer cell death by enhancing ROS production and modulating the immune response. However, few studies have investigated the antitumor effects of LAA, an MCFA, on CRCs. Therefore, in this study, we investigated the effects of LAA on tumor cells and host antitumor immunity in CRC cell lines.

Our results demonstrated that LAA treatment increased mitochondrial ROS production in cancer cells, leading to cell death. In contrast, in lymphocytes, LAA activated OXPHOS without inducing ROS production, thereby promoting the antitumor activity of CD8+ T lymphocytes. LAA treatment also showed a strong antitumor effect in a peritoneal dissemination model using syngeneic CT26 cells and BALB/c mice.

LAA promotes ROS production in cancer cells but not normal lymphocytes [20,21]. This difference may be due to impaired mitochondrial quality control in cancer cells caused by dysfunctional mitophagy and biogenesis [35,36]. As a result, various mitochondrial DNA mutations occur frequently in cancer cells [37], leading to imbalanced gene expression in the electron transport chain (ETC) in cancer cells [38,39], which suppresses the tricarboxylic acid cycle and OXPHOS and enhances ROS production [20,40].

LAA rapidly enters the mitochondria in a carnitine shuttle-independent manner to promote OXPHOS [41,42]. Thus, LAA may induce excessive ROS by forcing OXPHOS in the mitochondria with an imbalanced ETC complex expression, leading to cell death [20,39]. In contrast, no imbalance in mitochondrial gene expression has been observed in mouse peripheral blood lymphocytes, and LAA does not induce such an imbalance [20]. We previously reported that LAA exerts a protective effect on skeletal and cardiac muscles in tumor-bearing mice by suppressing cachexia [31,32]. However, when large amounts of LAA are administered, ROS levels also increase in the myocardium [43]. In normal cells, ROS production remains low compared with that in cancer cells, despite the promotion of OXPHOS. The qualitative difference in mitochondria between cancer cells and lymphocytes may lead to their different reactivities to LAA.

In the present study, LAA increased ROS levels and induced cell death in both CT26 and HT29 CRC cell lines, but induced cell death more strongly in HT29 cells than in CT26 cells. Cell death induced by oxidative stress includes apoptosis, ferroptosis, necrosis, pyroptosis, and autophagic cell death. In apoptosis, mitochondrial damage leads to the release of cytochrome C into the cytoplasm and activation of the caspase pathway from caspase-9 to -3/-7. We also observed differences in mitochondrial turnover between these cell lines. Mitochondrial turnover refers to the removal of defective mitochondria via mitophagy [44] and the biogenesis of newly formed mitochondria [45]. In the present study, LAA treatment increased the mitochondrial volume in CT26 cells but decreased it in HT29 cells. These results suggest decreased mitochondrial turnover in HT29 cells compared with CT26 cells, and that the increase in ROS from accumulated impaired mitochondria more strongly enhanced cell death in HT29 cells than in CT26 cells.

In addition, our data showed that LAA enhanced and suppressed cancer cell stemness in CT26 and HT29 cells, respectively. Unlike non-cancer stem cells, cancer stem cells have an OXPHOS-dominated metabolism [46,47]. In CT26 cells, LAA caused a greater reduction in mitochondrial membrane potential compared to HT29 cells, potentially promoting mitophagy to a greater extent. Nevertheless, the mitochondrial content was maintained in CT26 cells, whereas it decreased in HT29 cells. This suggests that mitochondrial turnover is more active in CT26 cells than in HT29 cells. The newly generated mitochondria may contribute to the enhancement of OXPHOS and stemness [48].

In the 3D co-culture of CT26 cells and SplCs, LAA suppressed sphere formation ability, suggesting that LAA exerts anti-cancer stem cell activity in vivo. Our data showed higher CD8α RNA levels in the tumor tissues of mice fed LAA compared with the control group, suggesting that LAA facilitated the intratumoral infiltration of CD8α-positive effector T lymphocytes. Lymphocyte infiltration is a favorable prognostic factor in cancer [49,50]. The LAA-induced infiltration of effector T lymphocytes into tumors may promote the antitumor effects of LAA in vivo. However, this finding requires further verification in future studies using animal models of metastatic cancer.

The results of the present study suggest that the effect of LAA on the energy metabolism of SplCs may lead to CD8α+ T cell activation. Various reports have described energy metabolism in immune cells. For instance, in plasmacytoid dendritic cells, type I IFN production is promoted by glycolysis, whereas antiviral responses depend on OXPHOS activity [51]. Additionally, CD4+ T cell proliferation and cytokine production depend on glutaminolysis [52]. Regulatory T cells require increased ROS and OXPHOS levels for forkhead box P3 expression, differentiation, and anti-inflammatory interleukin-10 cytokine synthesis [53]. In contrast, glycolysis is enhanced in effector T cells [54], and the suppression of glycolysis inhibits cytotoxic CD8 T cell effector responses [53]. However, effector T cells are exhausted and lose their activity in the presence of tumors [55,56]. Promotion of β-oxidation reactivates exhausted effector T cells [57], and OXPHOS enables their sustained activation [58].

ConA, used in our experiments, promotes mitochondrial respiration [59]. However, excessive proliferation stimulation may have caused T cell exhaustion in SplCs expressing programmed death-ligand-1, a marker of exhaustion [60]. The increased MCAD expression and mitochondrial membrane potential in LAA-treated SplCs suggested the promotion of β-oxidation and OXPHOS, which may have reactivated exhausted CD8α+ T cells. Reactivation of these exhausted effector T cells by LAA treatment may explain the enhanced antitumor effect of LAA in our in vitro experimental system. A similar reactivating effect of LAA on effector T cells is expected in human cancers.

LAA treatment enhanced *PGC-1α* expression in SplCs in the present study. PGC-1α is a mitochondrial biogenesis marker [61], the increased expression of which promotes memory T cell formation [62]. Memory T cells are mainly metabolized via OXPHOS and fatty acid oxidation [63], and ketone bodies epigenetically regulate memory T cell formation and maintenance [64]. LAA is efficiently metabolized in the liver to produce ketone bodies [65]. Thus, LAA promotes the differentiation and activation of both effector and memory T cells. These effects of LAA on memory T cells may be involved in the promotion of antitumor effects in the mouse model used in this study.

The energy metabolism of tumor cells, that is, the ratio of glycolysis to OXPHOS, changes the tumor microenvironment (TME) and affects antitumor immunity [66]. Upregulation of the mitochondrial OXPHOS pathway and inhibition of glycolysis not only reduce lactate secretion from tumor cells and promote intratumoral infiltration and IFN-γ secretion by CD8+ and CD4+ T cells but also suppress regulatory T cells and myeloid-derived suppressor cells, resulting in excellent antitumor effects [66]. In contrast, decreased lactate levels in the TME may result in an OXPHOS-inhibitory effect due to decreased lactate uptake by lymphocytes [67]. Additionally, decreased glucose uptake due to decreased glycolysis in cancer cells may increase glucose concentration in the TME and promote glycolysis in CD8+ T cells through the Crabtree effect [68]. We did not observe cytotoxicity in the co-culture of cancer cells and SplCs without LAA. However, treatment of the co-culture system with LAA enhanced the invasion of SplCs into spheres and cytotoxicity in the 2D culture system. These findings suggest that the antitumor effect of LAA may reflect changes in the TME owing to alterations in the energy metabolism of tumor cells.

There are several limitations in this study. We examined the effects of LAA on T cells and discussed their differentiation into subtypes. However, we did not investigate antigen-specific activation of LAA or quantitative investigation of T cell subtypes; therefore, future flow cytometry-based studies are required. Only one type of mouse or two CRC cell lines are considered in a modest number. A more extensive study with a large number of mice of multiple species or various cell lines is needed. Identifying understudied areas and new aspects of LAA action, investigating specific molecular targets and signal transduction pathways of LAA, and the effects of LAA in combination with other new therapeutic agents will be examined and clarified in the future, which will advance LAA research.

The in vitro and in vivo results of the present study demonstrated the excellent antitumor effects of LAA. One potential mechanism is the induction of cell death associated with increased ROS production in cancer cells and the induction of cytotoxic activity by reactivating immune cells, particularly CD8α+ T cells. LAA is a widely used food nutrient. It can be used alone as an anti-cancer drug, but it is expected that it will be useful in promoting antitumor effects and reducing side effects when combined with existing antitumor treatments. It is also expected to be effective in preventing recurrence through long-term administration. In addition, the safety and appropriate dosage of LAA will also need to be considered. Therefore, active clinical research on the application of LAA in cancer treatment is warranted.

## 4. Materials and Methods

### 4.1. Cell Lines and Reagents

HT29 human carcinoma cells were purchased from Dainihon Pharmacy Co. (Tokyo, Japan), while CT26 murine colon carcinoma cells were kindly provided by Professor I. J. Fidler (MD Anderson Cancer Center, Houston, TX, USA). Cells were cultured in DMEM with 10% FBS at 37 °C in 5% CO_2_ and treated with LAA (40 μg/mL, IC_20_). Cell line authentication by short tandem repeat profiling was performed before starting this study (Takara Bio, Kyoto, Japan). Apoptosis was assessed by counting apoptotic bodies in 1000 Giemsa-stained cells (Sigma-Aldrich, St. Louis, MO, USA).

### 4.2. Fluorescent Labeling of LAA

To visualize intracellular localization, LAA was fluorescently labeled with 9-anthryldiazomethane (ADAM; Funakoshi, Tokyo, Japan), which forms strongly fluorescent esters upon reacting with fatty acids. A 0.1% ADAM solution was prepared by dissolving ADAM (1 mg) in methanol (1 mL). LAA (100 µL) was mixed with the ADAM solution (100 µL) in the dark at room temperature for 2 h. CT26 cells were then treated with labeled LAA and Mitogreen (100 nM, Molecular Probes, Eugene, OR, USA) for mitochondrial visualization. Fluorescence images were captured using a BZ-X700 microscope (KEYENCE, Osaka, Japan).

### 4.3. MTS [3-(4,5-Dimethylthiazol-2-yl)-5-(3-carboxymethoxyphenyl)-2-(4-sulfophenyl)-2H-tetrazolium] Assay

MTS assays were conducted using the CellTiter 96 Aqueous One Solution Cell Proliferation Assay Kit (Promega Biosciences Inc., San Luis Obispo, CA, USA). Absorbance was measured at 490 nm using a Multiskan FC microplate photometer (Thermo Fisher Scientific, Waltham, MA, USA). The MTS value of cells treated with the control oligonucleotide served as the reference.

### 4.4. Mitochondrial Imaging

Mitochondrial function was assessed using fluorescent probes. After treatment with or without LAA (40 μg/mL), cells were incubated with the probes for 30 min at 37 °C and imaged using an all-in-one fluorescence microscope (KEYENCE). The following probes were used: DHR123 (100 μM, Dojindo, Kumamoto, Japan) for mitochondrial H_2_O_2_, MitoSOX (10 μM, AAT Bioquest Inc., Sunnyvale, CA, USA) for mitochondrial superoxide and oxidative stress, MitoGreen (100 nM, PromoCell GmbH, Heidelberg, Germany) for mitochondrial volume, and TMRE (200 nM, Sigma-Aldrich) for mitochondrial membrane potential.

### 4.5. Protein Extraction

Whole-cell lysates were prepared as previously described using radioimmunoprecipitation assay buffer with 0.1% sodium dodecyl sulfate (Thermo Fisher Scientific, Tokyo, Japan) [69]. Protein concentrations were measured using a Protein Assay Rapid Kit (Wako Pure Chemical Corporation, Osaka, Japan).

### 4.6. Enzyme-Linked Immunosorbent Assay (ELISA) and Fluorometric Assay

An ELISA kit was used to measure the concentrations of 4HNE, lactate, granzyme B, TNF-α, IFN-γ, and CD8α in whole-cell lysates (Table 2), following the manufacturer’s instructions.

### 4.7. Sphere Assay

Cells (1000 cells/well) were seeded in uncoated 35 mm bacteriological dishes (Corning Inc., Corning, NY, USA) with 3D Tumorsphere Medium XF (Sigma-Aldrich) and cultured with or without LAA (40 μg/mL). After 7 days, sphere images were captured digitally, and sphere numbers were analyzed using NIH ImageJ software (version 1.52, Bethesda, MD, USA).

### 4.8. Mitochondrial Stress Test (Seahorse Assay)

Mitochondrial respiration and ATP production were analyzed using a Seahorse XF Analyzer (Agilent Technologies, Santa Clara, CA, USA) to measure extracellular flux in live cells. Following treatment (40 µg/mL, 48 h), cells were collected, seeded into an XF plate at 2 × 10^4^ cells/well, and incubated overnight. The following day, the medium in the XF plate was replaced with XF DMEM, 1 h before the assay. The Mito Stress Test (Seahorse XF Cell Mito Stress Test, Agilent) was performed according to the manufacturer’s protocol. OCR was measured under the following conditions: 2 µM oligomycin, 0.5 µM carbonyl cyanide-p-trifluoromethoxyphenylhydrazone, and 0.5 µM rotenone/antimycin A. OCR values were normalized to total cellular protein concentration, determined after protein extraction from the analyzed cells.

### 4.9. Animals

Five-week-old male BALB/c mice were purchased from SLC (Shizuoka, Japan) and housed in a pathogen-free facility under a 12-h light/dark cycle at 22 °C in a humidity-controlled environment at the Animal Laboratory in Nara Medical University. The study was conducted in accordance with institutional guidelines approved by the Committee for Animal Experimentation of Nara Medical University, Kashihara, Japan, following the regulations and standards of the Japanese Ministry of Health, Labour and Welfare (approval no. 12262). The animals were acclimated to their housing for seven days before the experiment began. Accordance with institutional guidelines was approved by the Committee for Animal Experimentation of Nara Medical University, Kashihara, Japan, following current regulations and standards of the Japanese Ministry of Health, Labour and Welfare (approval no. 12262). The animals were allowed to acclimate to their housing for seven days before the start of the experiment. The mice were fed a CE-2 diet (CLEA Japan, Inc., Tokyo, Japan) or an LAA diet (CE-2 diet mixed with 2% *w*/*w* LAA) (Table 1). For peritoneal dissemination, CT26 cancer cells (5 × 10^6^ cells) were inoculated into the peritoneal cavity of syngeneic BALB/c mice (n = 5). The condition and weight of the mice were monitored daily. The mice were euthanized under sevoflurane anesthesia (Maruishi Pharmaceutical, Osaka, Japan) two weeks after inoculation. Tumor weight was measured by dissecting peritoneal tumors from the intestine, mesentery, diaphragm, and abdominal wall, with non-tumoral tissues grossly removed.

### 4.10. RT-PCR

To assess human and murine mRNA expression, RT-PCR was performed using 0.5 µg of total RNA extracted from both cell lines with an RNeasy kit (Qiagen, Germantown, MD, USA). Primer sets (listed in Table 2) were synthesized by Sigma Genosys (Ishikari, Japan). PCR products were electrophoresed on a 2% agarose gel and stained with ethidium bromide. Glyceraldehyde-3-phosphate dehydrogenase mRNA was amplified as an internal control.

### 4.11. Spleen Cell Isolation

The spleens of 5-week-old male BALB/c mice were minced in 5 mL of Hanks’ balanced salt solution (HBSS, Wako) using a scalpel. A DNase solution (20 μg/mL, Sigma) containing 1% FBS (Wako) was added, and the cells were incubated at 37 °C for 30 min. Enzymatic reactions were halted by adding 1 mM ethylenediaminetetraacetic acid (EDTA; Wako). The cell suspension was filtered through a 0.22 μm filter (Merck, Tokyo, Japan) and washed three times with 10 mL of phosphate-buffered saline (PBS). It was then centrifuged at 500× *g* for 5 min at 4 °C, and the supernatant was discarded. Red blood cells were lysed using a red blood cell lysis buffer (Funakoshi, Tokyo, Japan). The remaining spleen cells were centrifuged again at 500× *g* for 5 min at 4 °C and resuspended in cold PBS at a concentration of 1 × 10^8^ cells/mL.

### 4.12. Cell Surface Labeling

Spleen and CT26 cells were surface-labeled with PKH26green and PKH26red, respectively (Sigma-Aldrich) for in vivo cell-tracking. PKH26 does not inhibit stem cell proliferation or cause stem cell toxicity [70]. The cell suspensions were mixed with an equal volume of labeling solution containing 4 μM PKH26 in dilution buffer and incubated at room temperature for 5 min. The reaction was stopped by adding 2 mL of FBS, followed by three washes with DMEM before further experiments.

### 4.13. Statistical Analysis

Statistical significance was assessed using a two-tailed Fisher’s exact test and ordinary analysis of variance (ANOVA) with InStat software v3.1 (GraphPad, San Diego, CA, USA). A two-sided *p* < 0.05 was considered statistically significant. All experimental designs were based on a space-filling design.

## 5. Conclusions

LAA treatment increased mitochondrial ROS production in cancer cells, ultimately leading to cell death. In contrast, in host lymphocytes, LAA activated OXPHOS without inducing ROS production, thereby enhancing the antitumor activity of CD8+ T lymphocytes. Through this dual mechanism—directly inducing cancer cell death and boosting the immune response—LAA exhibited significant antitumor effects. As a naturally occurring dietary nutrient that is safely consumed, LAA holds promise for clinical application, either as a standalone antitumor agent or in combination with existing anti-cancer therapies.

## Figures and Tables

**Table 1:** Diet ingredients.

Ingredient	Diets	
	CE-2	LAA
LAA (%)	-	2
Moisture (%)	8.83	8.6534
Crude protein (%)	25.13	24.6274
Crude fat (%)	4.92	4.8216
Crude fiber (%)	4.42	4.3316
Crude ash (%)	6.86	6.7228
NFE (%)	49.84	48.8432
Energy (kcal)	344.2	355.316

LAA, lauric acid; NEF, nitrogen free extract.

**Table 2:** Primer sets and ELISA kits.

RT-PCR Primers				
Gene	Species	ID	Left	Right
*ACTB*	Mouse	NM_007393.5	AGCCATGTACGTAGCCATCC	CTCTCAGCTGTGGTGGTGAA
*ACTB*	Human	NM_001101.3	GGACTTCGAGCAAGAGATGG	AGCACTGTGTTGGCGTACAG
*CD44*	Mouse	M27130.1	TGGATCCGAATTAGCTGGAC	AGCTTTTTCTTCTGCCCACA
*CD44*	Human	FJ216964.1	AAGGTGGAGCAAACACAACC	AGCTTTTTCTTCTGCCCACA
*CD133*	Mouse	BC028286.1	GAAAAGTTGCTCTGCGAACC	TCTCAAGCTGAAAAGCAGCA
*CD133*	Human	BC012089.1	TTGTGGCAAATCACCAGGTA	TCAGATCTGTGAACGCCTTG
*LGR5*	Mouse	NM_010195.2	CATTCACTTTTGGCCGTTTT	AGGGCCAACAGGACACATAG
*LGR5*	Human	AF061444.1	CTCTTCCTCAAACCGTCTGC	GATCGGAGGCTAAGCAACTG
*MCAD*	Mouse	NM_000016.6	AAATCATCCCAGTGGCTGCA	ACATCGCTGGCCCATGTTTA
*PGC1A*	Mouse	BC066868.1	ATGTGTCGCCTTCTTGCTCT	ATCTACTGCCTGGGGACCTT
*CMYC*	Mouse	AH005318.2	GCCCAGTGAGGATATCTGGA	ATCGCAGATGAAGCTCTGGT
*Ki67*	Mouse	X82786.1	GACAGCTTCCAAAGCTCACC	TGTGTCCTTAGCTGCCTCCT
*NS*	Mouse	BC037996.1	ATGTGGGGAAAAGCAGTGTC	TGGGGGAGTTACAAGGTGAG
ELISA				
Target	Species	Cat#	Manufacturer	
4HNE	-	ab287803	Abcam, Waltham, MA, USA	
Lactate	-	ab65331	Abcam, Waltham, MA, USA	
Granzyme B	Mouse	#88-8022-88	Thermo Fisher, Tokyo, Japan	
TNF-α	Mouse	#A43658	Thermo Fisher, Tokyo, Japan	
IFN-γ	Mouse	#A100150	Thermo Fisher, Tokyo, Japan	
CD8α	Mouse	PO1731	RayBiotech, Peachtree Corners, GA, USA	

ELISA, enzyme-linked immunosorbent assay; RT-PCR, reverse transcription-polymerase chain reaction; ACTB, β-actin; LGR5, leucine-rich repeat-containing G-protein coupled receptor 5; MCAD, medium-chain acyl-CoA dehydrogenase; PGC1A, peroxisome proliferator-activated receptor gamma coactivator 1-alpha; NS, nucleostemin; 4HNE, 4-hydroxynonenal; TNF, tumor necrosis factor; IFN, interferon.

## Data Availability

Data is contained within the article.

## Abbreviation

LAA	lauric acid
CRC	colorectal cancer
ROS	reactive oxygen species
OXPHOS	oxidative phosphorylation
LCFA	long-chain fatty acid
MCFA	medium-chain fatty acid
TMRE	tetramethylrhodamine ethyl ester
DHR123	dihydrorhodamine 123
4HNE	4-hydroxynonenal
OCR	oxygen consumption rate
LGR5	leucine-rich repeat-containing G-protein coupled receptor 5
RT-PCR	reverse transcription-polymerase chain reaction
ConA	concanavalin A
TNF	tumor necrosis factor
IFN	interderon
MCAD	medium-chain acyl-CoA dehydrogenase
PGC-1α	peroxisome proliferator-activated receptor gamma coactivator-1α
NS	nucleostemin
ETC	electron transport chain
SplC	spleen cell
TME	tumor microenvironment
